# The association between 38 previously reported polymorphisms and psoriasis in a Polish population: High predicative accuracy of a genetic risk score combining 16 loci

**DOI:** 10.1371/journal.pone.0179348

**Published:** 2017-06-15

**Authors:** Bartłomiej Kisiel, Katarzyna Kisiel, Konrad Szymański, Wojciech Mackiewicz, Ewelina Biało-Wójcicka, Sebastian Uczniak, Anna Fogtman, Roksana Iwanicka-Nowicka, Marta Koblowska, Helena Kossowska, Grzegorz Placha, Maciej Sykulski, Artur Bachta, Witold Tłustochowicz, Rafał Płoski, Andrzej Kaszuba

**Affiliations:** 1 Department of Internal Diseases and Rheumatology, Military Institute of Medicine, ul. Szaserów 128, Warszawa, Poland; 2 Department of Dermatology, Pediatric and Oncologic Dermatology, Medical University of Łódź, ul. Kniaziewicza 1/5, Łódź, Poland; 3 Department of Pediatric Dermatology, Center of Dermatology, Międzyleski Specialist Hospital, ul. Bursztynowa 2, Warszawa, Poland; 4 Department of Medical Genetics, Medical University of Warsaw, ul. Pawińskiego 3c, Warszawa, Poland; 5 Department of Dermatology, Medical University of Warsaw, ul. Koszykowa 82a, Warszawa, Poland; 6 Department of Dermatology, Center of Dermatology, Międzyleski Specialist Hospital, ul. Bursztynowa 2, Warszawa, Poland; 7 Institute of Biochemistry and Biophysics, Polish Academy of Sciences, ul. Pawińskiego 5a, Warszawa, Poland; 8 Laboratory of Systems Biology, Faculty of Biology, University of Warsaw, ul. Pawińskiego 5a, Warszawa, Poland; 9 Department of Internal Medicine, Hypertension, and Vascular Diseases, Medical University of Warsaw, ul. Banacha 1a, Warszawa, Poland; 10 Department of Medical Informatics and Telemedicine, Medical University of Warsaw, ul. Banacha 1a, Warszawa, Poland; South Texas Veterans Health Care System, UNITED STATES

## Abstract

**Objectives:**

To confirm the association of previously discovered psoriasis (Ps) risk loci with the disease in a Polish population and to create predictive models based on the combination of these single nucleotide polymorphisms (SNPs).

**Material and methods:**

Thirty-eight SNPs were genotyped in 480 Ps patients and 490 controls. Alleles distributions were compared between patients and controls, as well as between different Ps sub-phenotypes. The genetic risk score (GRS) was calculated to assess the cumulative risk conferred by multiple loci.

**Results:**

We confirmed associations of several loci with Ps: *HLA-C*, *REL*, *IL12B*, *TRIM39*/*RPP21*, *POU5F1*, *MICA*. The analysis of ROC curves showed that GRS combining 16 SNPs at least nominally (uncorrected *P*<0.05) associated with Ps (GRS-N) had significantly better discriminative power than GRS combining SNPs associated with Ps after the Bonferroni correction (AUC 0.776 vs. 0.750, *P* = 1 x 10^−4^) or *HLA-*C (AUC 0.776 vs. 0.694, *P*<1 x 10^−5^). On the other hand, adding additional SNPs to the model did not improve its discriminatory ability (AUC 0.782 for GRS combining all SNPs, *P*>0.05). In order to assess the total risk conferred by GRS-N, we calculated ORs according to GRS-N quartile ˗ the Ps OR for top vs. bottom GRS-N quartiles was 12.29 (*P*<1 x 10^−6^). The analysis of different Ps sub-phenotypes showed an association of GRS-N with age of onset and family history of Ps.

**Conclusions:**

We confirmed the association of Ps with several previously identified genetic risk factors in a Polish population. We found that a GRS combining 16 SNPs at least nominally associated with Ps had a significantly better discriminatory ability than *HLA-*C or GRS combining SNPs associated with Ps after the Bonferroni correction. In contrast, adding additional SNPs to GRS did not increase significantly the discriminative power.

## Introduction

Psoriasis (Ps) is a chronic inflammatory disease of the skin affecting 2–3% of Caucasians [1]. About 75% of patients develop Ps before the age of 40 years ˗ early-onset or type I Ps (T1Ps). Type II Ps (T2Ps) or late-onset Ps is observed after the age of 40 years [2]. About 30% of Ps patients develop inflammatory arthritis (psoriatic arthritis, PsA) [3,4]. Nail Ps is seen in about 50% of patients at Ps diagnosis, with a lifetime incidence reaching 80–90% [5].

The pathogenesis of Ps is complex involving both environmental and genetic factors. Environmental factors include smoking, streptococcal infection (for acute guttate Ps), stress, drugs (beta-blockers, interferon, anti-malarials, lithium), cold weather, diet, and obesity [6]. Epidemiological studies provide evidence of genetic contributions to the development of Ps, with a higher incidence of the disease in first- and second-degree relatives of patients than in general population [7]. Furthermore, concordance rates are higher for monozygotic twins than for dizygotic twins (35–72% vs. 12–23%) [8]. Linkage and association studies demonstrated that the MHC region harbors the major genetic determinant for Ps susceptibility (PSORS1). HLA-Cw*0602 is the most likely susceptibility allele in this locus, accounting for 35–50% of disease heritability [9]. Further candidate gene association studies and genome wide association studies (GWAS) identified several susceptibility loci both within and outside MHC. At present, about 40 additional loci are thought to be associated with Ps [10]. Genes corresponding to these loci are involved in the key pathogenesis pathways including: epidermal differentiation pathway (*LCE3B*, *LCE3C*), IL-12/IL-23 pathway (*IL12*, *IL23A*, *IL23R*, *TRAF3IP2*, *TYK2*), NFκB and IFN signaling pathway (*TNFAIP3*, *TNIP1*, *NFKBIA*, *REL*, *TYK2*, *IFIH1*, *IL23RA*), Th2 pathway (*IL4*, *IL13*) and adaptive immunity involving CD8 T cells (*ERAP1*, *ZAP70*) [11]. The underlying genetics may determine the age of onset as well as the disease course. T1Ps has been shown to have a higher degree of heritability and a higher prevalence of HLA-Cw*0602 [2,12]. The presence of the risk allele HLA-Cw*0602 was reported to be associated with a more severe disease course and a higher prevalence of the guttate phenotype, while nail Ps and PsA were shown to be more common in HLA-Cw*0602-negative patients [12,13]. It should be emphasized that the genetic contribution to PsA risk is less understood than to Ps. On the one hand, a higher recurrence risk ratio in the first-degree relatives in PsA as compared to Ps (30–55 for PsA vs. 5–10 in Ps) suggests a higher genetic contribution to PsA [11,14]. On the other hand, nearly all PsA susceptibility loci identified by GWAS are also associated with Ps.

In the case of complex diseases (such as psoriasis) single genetic markers have limited impact on disease risk. Combining multiple loci with moderate effect into a genetic risk score (GRS) might improve identifying individuals with an increased risk for the disease [15,16]. This approach was shown to be effective in several complex traits, including type 2 diabetes mellitus, rheumatoid arthritis, multiple sclerosis, stroke and myocardial infarction [17–22]. Chen et al. showed that a GRS combining 10 psoriasis risk loci captured significantly more risk than any individual SNP and was associated with an early onset of disease and a positive family history [23]. Yin et al. found that a GRS combining 14 psoriasis susceptibility loci had a very good discriminating potential and was associated with family history and age of onset [24]. A recent study conducted in a population from Northern Poland showed that a panel of 5 susceptibility loci had higher accuracy for the disease prediction than any marker analyzed separately [25].

In this study we aimed to replicate the association of Ps with 39 previously reported single nucleotide polymorphisms (SNPs). We also sought to create predictive models based on the combination of these SNPs and to evaluate their discriminatory performance in a large cohort of Polish patients.

## Materials and methods

The study was approved by the Medical University of Łódź Ethics Committee. Written informed consent was obtained from each patient. All procedures were performed in accordance with the Helsinki Declaration of 1975, as revised in 1983.

### Patients and controls

The study group consisted of 480 psoriasis patients. The patients were recruited at the Medical University of Łódź Department of Dermatology, Pediatric and Oncologic Dermatology (Łódź, Poland), Medical University of Warsaw Department of Dermatology (Warsaw, Poland) and Międzyleski Specialist Hospital Center of Dermatology Department of Dermatology (Warsaw, Poland). The inclusion criterion was a clinical diagnosis of psoriasis vulgaris established by an experienced dermatologist. The exclusion criteria were: (i) another (than psoriasis vulgaris) type of Ps, (ii) another coexisting autoinflammatory disease, (iii) history of malignancy. A short structured questionnaire was used to collect data regarding age, gender, age at Ps onset, nail involvement, history of PsA (confirmed by a rheumatologist) and family history of Ps. Each patient was examined by an experienced dermatologist. Psoriasis Area and Severity Index (PASI) was used to assess disease severity [26,27]. Patients were classified as T1Ps or T2Ps (disease onset <40 and ≥40 years of age, respectively). The abbreviation PsC refers to individuals with purely cutaneous Ps (without joint involvement) and PsA to patients with psoriatic arthritis (all PsA patients had also skin involvement). The control group comprised 490 anonymous unrelated individuals matched for sex and ethnicity, selected from a repository used in previous studies and consisted mainly of individuals requesting paternity testing.

### Single nucleotide polymorphisms (SNPs) selection

For this study we selected SNPs previously associated with Ps and/or PsA [28–42]. The criteria for SNPs selection were as follows: (i) association confirmed in GWA, meta-analysis or large scale case-control study, (ii) OR≥1.15 in at least one study, (iii) minor allele frequency (MAF) ≥0.10 in Caucasian population (based on data from HapMap CEU population). In general, 1 SNP was chosen for 1 locus, except for *IL23R*, *IL12B* and *HLA-C*. *IL23R* (rs7530511, rs11209026) and *IL12B* (rs3212227, rs6887695) SNPs were shown to be independent and to form common risk haplotypes [29]. The rs4406273 was found to be in very strong linkage disequilibrium (LD) with HLA-Cw*0602 in four populations of European descent from the United States, Finland, Great Britain, and Italy (r^2^ = 0.984), and in three Asian populations from Japan and China (r^2^ = 1.000); thus, it can be used as a substitute for HLA-Cw*0602 genotyping [43]. However, some studies used rs10484554 as a tag SNP for *HLA-C* [23] and thus, this SNP was also included. The rs3751385 and rs9304742 were shown to be associated with Ps in a Chinese population (both with OR = 1.14); however, a replication in a German population yielded ORs>1.15 (1.19 and 1.26, respectively) [35]. Additionally, 2 SNPs associated with multiple autoinflammatory diseases were included: rs10865331 (associated with Ps and ankylosing spondylitis) [44] and rs1250546 (associated with Ps and Crohn disease) [40]. Eventually, 39 SNPs were selected for genotyping (S1 Table).

### Genotyping

DNA was isolated from whole-blood samples using a salting-out method [45]. SNPs were genotyped using a GoldenGate (Illumina, CA, USA) custom assay according to the manufacturer's standard protocols. The genotyping success rate was >98% for all SNPs. One of the genotyped SNP (rs125046) was excluded from the analysis because of Hardy-Weinberg equilibrium deviation (*P* = 0.006).

### Statistical analysis

The PLINK statistical software package was used to evaluate the differences in allele frequencies of each SNP between cases and controls and to test the Hardy-Weinberg equilibrium (HWE) [46]. *P*<0.05 was considered as a significant deviation from the HWE. Other statistical analyses were performed using Statistica 12 package (StatSoft Inc). The Bonferroni correction (with a correction factor derived from the number of SNPs tested) was used to adjust for multiple testing. The genetic risk score (GRS) was calculated to assess the cumulative risk conferred by multiple loci. GRS was computed as a number of risk alleles multiplied by the natural logarithm of the odds ratio associated with each individual SNP. Because of the missing data 11 cases were excluded from the GRS analysis. As the rs4406273 and rs10484554 are in strong LD (r^2^ = 0.79) only rs4406273 was included in the GRS. We calculated the following GRSs: GRS-ALL (GRS combining all 38 SNPs), GRS-0.1 (GRS combining 19 SNPs associated/with a trend toward an association with psoriasis in our cohort, i.e. with a *P* value <0.1), GRS-N (GRS combining 16 SNPs at least nominally associated with Ps in our cohort, i.e. with uncorrected *P* value <0.05), GRS-B (GRS combining 6 SNPs which remained significantly associated with Ps after the Bonferroni correction), GRS-HLA (GRS including only rs4406273- a proxy for HLA-Cw*0602), GRS-N(+)HLA(-) (GRS-N without rs4406273) and GRS-N(subst.) (GRS-N with rs4406273 substituted by rs10484554). The SNPs forming particular GRSs are summarized in S2 Table. The GRS was stratified into quartiles for examination of a dose dependent effect. As previous studies used Ps OR for top vs. bottom GRS quartiles to evaluate the effect size of the association [23,24], we used a similar method to allow the present vs. previous studies to be compared. In order to compare the discriminative ability of different GRSs, we constructed receiver operating characteristic (ROC) curves and measured the area under the curve (AUC). The AUCs were compared using DeLong’s method. We used logistic regression to assess the phenotypic variation covered by GRS as well as to examine the relationship between GRS and Ps sub-phenotypes. To address the issue of overfitting, we conducted an internal validation of our top GRS (i.e. GRS-N) by randomly dividing the cohort population into 2 unequal (75% − Training Set and 25% − Test Set) groups. We used the larger group (Training Set) to rebuild the same model, which was then tested on the second group (Test Set).

## Results

Demographic and clinical characteristics of the patients are presented in Table 1. SNPs associations with Ps are presented in Table 2.

**Table 1:** Demographic and clinical characteristics of the patients.

	Value
**Sex, male/female**	259/221
**Disease onset, mean (SD), years**	26.27 (16.32)
**Nail psoriasis**	251 (52.29%)
**Psoriatic arthritis**	162 (33.75%)
**PASI, mean (SD)**	16.65 (11.59)
**Family history of psoriasis**	208 (43.33%)

PASI- Psoriasis Area and Severity Index

**Table 2:** SNPs associations with psoriasis.

SNP	Chromosomal localization	Gene	Risk allele	RAF_ctrl_	RAF_cases_	OR (95% CI)	*P*
**rs7552167**	1p36.11	*IL28RA*	G	0.833	0.871	1.36 (1.05–1.75)	**0.017**
**rs7530511**	1p31.3	*IL23R*	C	0.851	0.866	1.13 (0.87-1-46)	0.37
**rs11209026**	1p31.3	*IL23R*	G	0.960	0.979	1.98 (1.14–3.44)	**0.013**
**rs2476601**	1p13.2	*PTPN22*	G	0.859	0.869	1.10 (0.85–1.44)	0.46
**rs4112788**	1q21.3	*LCE3C-LCE3B*	C	0.627	0.667	1.19 (0.99–1.44)	0.07
**rs6701216**	1q21.3	*LCE1C*	T	0.147	0.168	1.18 (0.92–1.51)	0.20
**rs702873**	2p13-p12	*REL*	G	0.557	0.642	1.42 (1.18–1.71)	**2.5 x 10**^**−4**^
**rs10865331**	2p15	*B3GNT2*	A	0.400	0.467	1.32 (1.10–1.60)	**2.5 x 10**^**−3**^
**rs17716942**	2q24	*IFIH1*	T	0.893	0.898	1.07 (0.79–1.43)	0.68
**rs30187**	5q15	*ERAP1*	T	0.313	0.344	1.15 (0.95–1.40)	0.14
**rs20541**	5q31.1	*IL13*	C	0.739	0.782	1.27 (1.02–1.56)	**0.026**
**rs1024995**	5q33.1	*TNIP1*	C	0.137	0.166	1.26 (0.98–1.62)	0.07
**rs3212227**	5q33.3	*IL12B*	A	0.774	0.853	1.69 (1.34–2.14)	**1.2 x 10**^**−5**^
**rs6887695**	5q33.3	*IL12B*	G	0.697	0.760	1.38 (1.12–1.69)	**2.1 x 10**^**−3**^
**rs2431697**	5q33.3	*PTTG1*	C	0.396	0.430	1.15 (0.96–1.38)	0.13
**rs6908425**	6p22.3	*CDKAL1*	C	0.775	0.811	1.24 (0.99–1.55)	0.051
**rs1150735**	6p21.3	*RNF39*	T	0.344	0.367	1.10 (0.91–1.33)	0.31
**rs1264569**	6p21.3	*TRIM39/RPP21*	A	0.794	0.871	1.75 (1.37–2.24)	**1.1 x 10**^**−5**^
**rs879882**	6p21.31	*POU5F1*	C	0.615	0.723	1.63 (1.35–1.98)	**3.4 x 10**^**−7**^
**rs4406273**	6p21.33	*HLA-C*	A	0.112	0.335	3.98 (3.13–5.06)	**4.6 x 10**^**−33**^
**rs10484554**	6p21.33	*HLA-C*	T	0.222	0.444	2.80 (2.29–3.41)	**5 x 10**^**−26**^
**rs13437088**	6p21.33	*MICA*	T	0.303	0.404	1.57 (1.30–1.90)	**3.3 x 10**^**−6**^
**rs240993**	6q21	*TRAF3IP2*	T	0.296	0.319	1.11 (0.92–1.35)	0.28
**rs610604**	6p23.3	*TNFAIP3*	C	0.305	0.325	1.09 (0.90–1.33)	0.37
**rs7007032**	8p23.2	*CSMD1*	T	0.690	0.691	1.00 (0.82–1.22)	0.99
**rs12580100**	12q13	*RPS26*	A	0.839	0.856	1.14 (0.89–1.46)	0.31
**rs3751385**	13q11-q12	*GJB2*	C	0.833	0.843	1.07 (0.84–1.37)	0.57
**rs7993214**	13q13.3	*COG6*	C	0.622	0.628	1.03 (0.85–1.24)	0.79
**rs8016947**	14q13	*NFKBIA*	G	0.531	0.591	1.27 (1.06–1.53)	**7.5 x 10**^**−3**^
**rs4780355**	16p13.13	*SOCS1*	T	0.668	0.680	1.06 (0.87–1.28)	0.58
**rs12445568**	16p11.2	*FBXL19*	C	0.406	0.427	1.09 (0.91–1.31)	0.35
**rs4795067**	17q11.2	*NOS2*	G	0.347	0.392	1.21 (1.01–1.46)	**0.049**
**rs744166**	17q21.31	*STAT3*	C	0.370	0.371	1.00 (0.83–1.21)	0.97
**rs12720356**	19p13.2	*TYK2*	T	0.929	0.951	1.49 (1.01–2.18)	**0.042**
**rs892085**	19p13.2	*IL3/CARM1*	T	0.602	0.634	1.15 (0.95–1.38)	0.15
**rs9304742**	19q13.41	*ZNF816*	C	0.320	0.339	1.09 (0.90–1.32)	0.36
**rs1008953**	20q12	*SDC4*	G	0.745	0.798	1.35 (1.09–1.68)	**5.9 x 10**^**−3**^
**rs2235617**	20q13.13	*RNF114*	G	0.522	0.568	1.20 (1.00–1.44)	**0.045**

CI- confidence interval; OR- odds ratio; RAF- risk allele frequency; *P* values <0.05 are in bold

As expected, the strongest association with Ps was found for SNPs in *HLA-C* locus: rs4406273 (OR = 3.98, *P* = 4.6 x 10^−33^) and rs10484554 (OR = 2.80, *P* = 5 x 10^−26^). These associations were stronger in T1Ps than in T2Ps (OR = 4.70, *P* = 3.3 x 10^−39^ vs. OR = 1.94, *P* = 1.6 x 10^−3^ for rs4406273 and OR = 3.19, *P* = 2.1 x 10^−30^ vs. OR 1.67, *P* = 2.3 x 10^−3^ for rs10484554). Several genotyped SNPs showed nominal association with Ps but only few SNPs remained significant after the Bonferroni correction: rs702873 (*REL*), rs3212227 (*IL12B*), rs1264569 (*TRIM39*/*RPP21*), rs879882 (*POU5F1*), rs13437088 (*MICA*). Most of these SNPs (except rs702873) showed an association with T1Ps (S3 Table). The only SNP to be significantly associated with T2Ps after the Bonferroni correction was rs13437088 (*HLA-C* SNPs showed only nominal association, S3 Table). The rs4406273 and rs10484554 were strongly associated with both PsC (OR = 4.09, *P* = 8.5 x 10^−29^, and OR = 2.84, P = 8.5 x 10^−29^) and PsA (OR = 3.79, *P* = 2.4 x 10^−18^, and OR = 2.69, P = 1.4 x 10^−13^). After the Bonferroni correction the rs702873, rs10865331, rs3212227, rs6887695, rs1264569, rs879882, rs13437088 showed an association with PsC, while rs1264569 ˗ with PsA (S4 Table).

**Fig 1:**
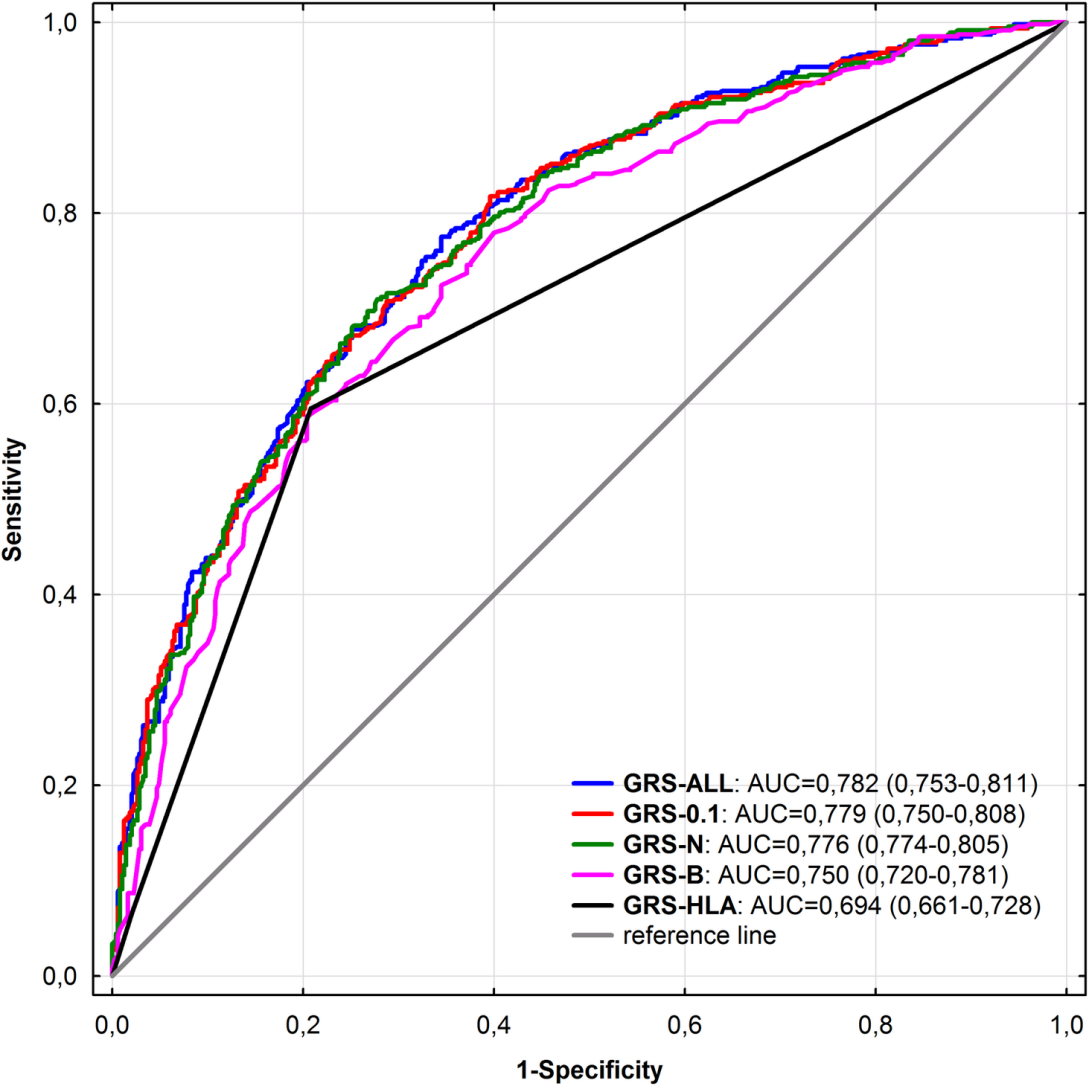
Comparison of ROC curves for prediction of psoriasis with the use of different genetic risk scores (GRS). GRS-ALL- GRS combining all 38 SNPs; GRS-0.1- GRS combining 19 SNPs associated/with a trend toward association with psoriasis; GRS-N- GRS combining 16 SNPs at least nominally associated with psoriasis in our cohort; GRS-B- GRS combining 6 SNPs which remained significantly associated with psoriasis after Bonferroni correction; GRS-HLA- GRS including only rs4406273 (a proxy for HLA-Cw*060). AUC- area under the curve.

The ROC curves for prediction of psoriasis with the use of GRSs are presented in Fig 1. The AUCs for GRS-ALL, GRS-0.1 and GRS-N were similar (0.782, 0.779 and 0.776, respectively) and the differences between them were insignificant. On the other hand, the AUC for GRS-N was significantly larger than AUC for GRS-B (0.776 vs. 0.750, *P* = 1 x 10^−4^) and AUC for GRS-HLA (0.776 vs. 0.694, *P*<1 x 10^−5^).

**Fig 2:**
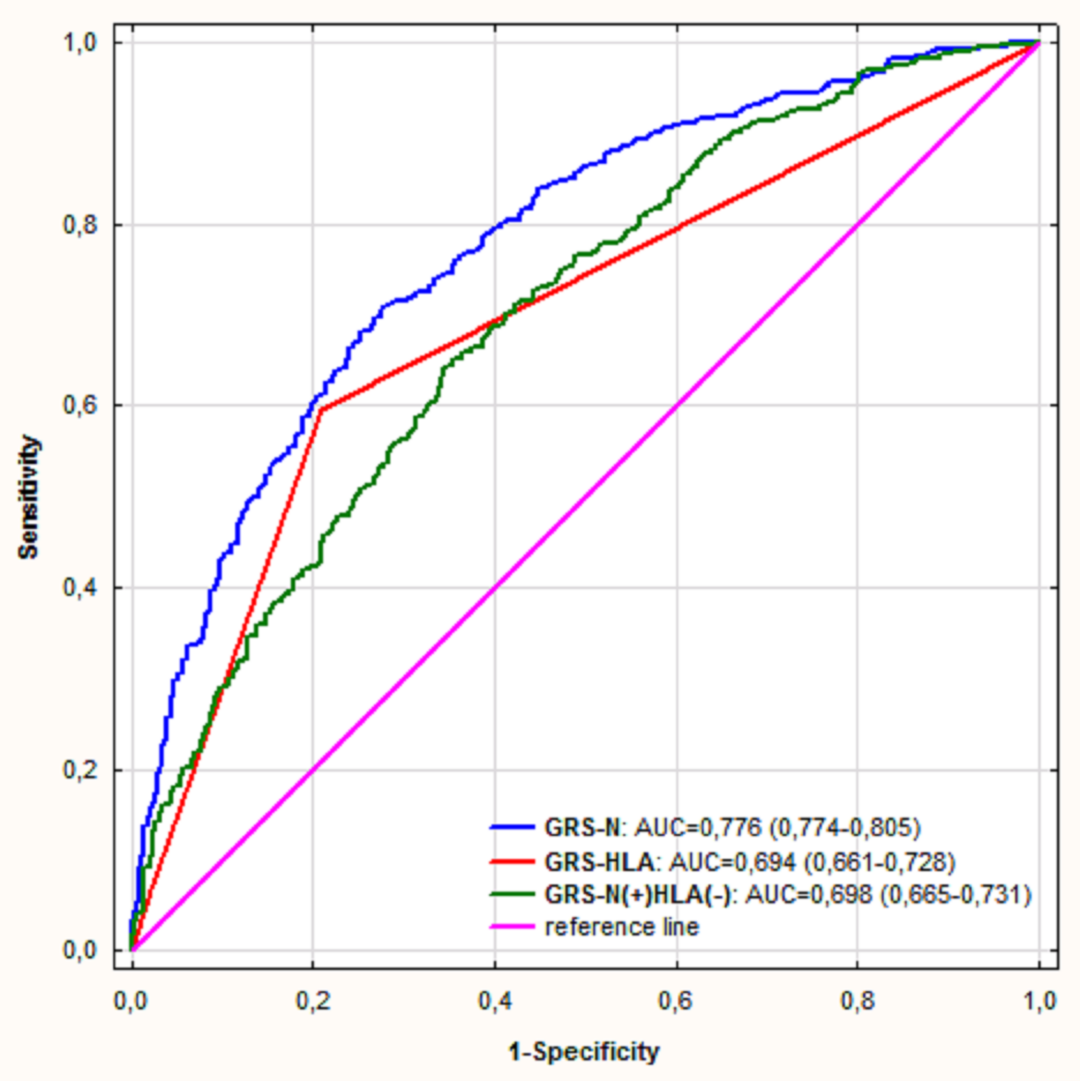
Comparison of ROC curves for prediction of psoriasis with the use of different genetic risk scores: GRS-N, GRS-HLA and GRS-N(+)HLA(-). GRS-N- GRS combining 16 SNPs at least nominally associated with psoriasis; GRS-HLA- GRS including only rs4406273 (a proxy for HLA-Cw*0602); GRS-N(+)HLA(-) (GRS-N without rs4406273). AUC- area under the curve.

To assess the discriminative power attributable to the *HLA-C* and non-*HLA-C* SNPs we compared the AUCs for GRS-N(+)HLA(-) and GRS-HLA (Fig 2), which were similar (0.694 vs. 0.698, *P* = 0.87) and significantly lower than AUC for GRS-N (*P*<1 x 10^−5^).

To assess the total risk conferred by the GRS-N, we calculated the ORs according to GRS-N quartile, using the first quartile as the reference group (Table 3). The Ps OR for top vs. bottom GRS-N quartiles was 12.29 (*P*<1 x 10^−6^). Interestingly, there were as many as 317 patients (67.2%) in the highest quartile category. In GRS logistic regression model we found that 16 SNPs forming GRS-N totally covered 19.63% phenotypic variation.

**Table 3:** The risk of psoriasis in GRS-N quartiles relative to the first quartile.

GRS-N	Number of cases	% of cases	Number of controls	OR (95% CI)	*P*
**< 3.10 (bottom Q)**	26	5.5	123		
**3.10–3.43**	39	8.3	122	1.51 (0.87–2.64)	0.14
**3.43–3.76**	90	19.1	122	3.49 (2.11–5.77)	<1 x 10^−6^
**> 3.76 (top Q)**	317	67.2	123	12.29 (7.67–19.70)	<1 x 10^−6^

95% CI- 95% confidence interval; OR- odds ratio; Q- quartile

We used logistic regression to assess the relationship between Ps sub-phenotypes and GRS-N (Table 4). GRS-N correlated negatively with age of onset and positively with family history of Ps.

**Table 4:** Association of GRS-N with psoriasis sub-phenotypes.

	Reference sub-phenotype	Test sub-phenotype	OR (95% CI)	*P*
**Nail psoriasis**	No	Yes	0.92	0.611
**Psoriatic arthritis**	No	Yes	0.73	0.089
**Age of onset**	<40 yrs of age	≥40 yrs of age	0.56	**0.014**
**Family history**	No	Yes	1.83	**0.006**

95% CI- 95% confidence interval; OR- odds ratio

We performed an internal validation of the GRS-N. The model showed a similar discriminatory ability (AUC) in Training vs. Test Sets (0.774 vs. 0.782, *P* = 0.805). The predictive performance of the GRS-N in both groups was assessed in terms of sensitivity, specificity, positive predictive value, negative predictive value and accuracy). The accuracy of the model in Training and Test Sets was similar (71.6% vs. 73.3%).

## Discussion

Psoriasis is a chronic inflammatory disease with a complex pathogenesis involving both genetic and environmental factors. Previous studies on multiply affected families have found several susceptibility loci for Ps [47]. The most strongly associated locus is on chromosome 6p21 within the MHC region (PSORS1) [48]. Family-based association studies have confirmed that *HLA-C* is directly involved in psoriatic susceptibility [49,50]. The HLA-Cw*0602 allele has been reported as a risk allele in numerous populations and is claimed to be associated with earlier disease onset, more severe disease course, and a higher prevalence of the guttate phenotype [12,13]. On the other hand, PsA and nail Ps have been reported to be more common in HLA-Cw*0602-negative patients [12]. We analyzed 2 SNPs in *HLA-C* locus: rs4406273 (almost perfect proxy for HLA-Cw*0602) and rs10484554. In our cohort the SNPs most strongly associated with Ps were rs4406273 and rs10484554 (OR = 3.98, *P* = 4.6 x 10^−33^ and OR = 2.80, *P* = 5 x 10^−26^). As expected, the association was stronger in T1Ps than T2Ps and in PsC than PsA.

We confirmed an association between Ps and rs702873 (*REL*), rs3212227 (*IL12B*), rs1264569 (*TRIM39*/*RPP21*), rs879882 (*POU5F1*), rs13437088 (*MICA*) in a Polish population. Further 10 SNPs showed a nominal association with Ps but became insignificant after the Bonferroni correction. We failed to find any association between Ps and the remaining 21 SNPs; however, at least in some cases, it may be due to the limited power of our study (S1 Table).

To assess the discriminatory ability of different GRSs we performed the ROC curves analysis. The comparison of AUCs for different GRSs showed that the predictive power increased gradually from GRS-HLA, through GRS-B to GRS-N (AUC = 0.694, 0.750 and 0.776, respectively). However, adding further SNPs (GRS-0.1 and GRS-ALL) did not improve significantly the discriminatory ability (AUC = 0.779 and 0.782, respectively).

GRS-N correlated negatively with age of onset and positively with family history of psoriasis. When we analyzed patients with T1Ps (mean age of onset 20.39 years) and those with T2Ps (mean age of onset 53.6 years) separately, we observed a significantly higher discriminatory ability of GRS-N in T1Ps (0.792 vs. 0.696). The ORs for top vs. bottom GRS-N quartiles were 16.06 (95% CI: 9.27–27.83, *P* = 4 x 10^−33^) in T1Ps and 4.56 (95% CI: 2.12–9.77, *P* = 3.5 x 10^−5^) in T2Ps patients. Similarly, the AUC for GRS-N was higher in patients with positive than in those with negative family history of Ps (0.814 vs. 0.747). Our results are supported by both previous studies analyzing the relationship between the GRS and Ps sub-phenotypes [23,24], which also demonstrated the association with age of onset and family history of Ps. Thus, these 2 factors (i.e. age of onset and family history) should be taken into account when comparing the predictive performance of different GRSs.

Few previous studies have reported good discriminatory ability of GRSs in Ps [23–25]. The similarities and differences between previous studies and our study are detailed in S6 Table.

Yin et al. described a GRS (further named GRS-Yin) combining 14 SNPs in a Han Chinese population [24]. Apart from the ethnicity, the study by Yin et al. differed from our study in terms of age of onset (21.31 years vs. 26.27 years) and proportion of patients with positive family history of Ps (31.34% vs. 43.3%). These differences make the direct comparison of the present study and that by Yin et al. questionable. The AUC for the GRS-Yin as well as OR for top vs. bottom GRS quartiles were markedly higher than in our study (0.8583 vs. 0.776 and 28.2 vs. 12.29, respectively). The SNPs forming GRS-Yin were found to cover 11.6% of phenotypic variation. It should be emphasized that the association between Ps and *HLA-C* was extremely strong in the study by Yin et al. (OR = 21.96 vs. 3.98 in our study) and most of the discriminative power of the GRS-Yin was attributable to *HLA-C* (AUC for *HLA*, non-*HLA* SNPs and *HLA* + non-*HLA* SNPs was 0.8343, 0.6029 and 0.8583, respectively), while in our study AUC for GRS-HLA was comparable to GRS-N(+)HLA(-) (0.694 vs. 0.698) and significantly lower than GRS-N (0.776). In other words, in our cohort adding 15 SNPs to *HLA-C* markedly increased the discriminatory ability, while in the Chinese cohort this gain was very modest. Thus, a better performance of the GRS-Yin was attributable to markedly larger effect size of the association between *HLA-C* and Ps. This may be simply due to different ethnicity but the difference in age of onset between the present study and that by Yin et al. should also be taken into account.

In a work published by Stawczyk-Macieja et al. conducted in a population of 294 individuals from Northern Poland (148 patients and 146 controls), a GRS (further named GRS-Stawczyk-Macieja) based on 5 markers showed a slightly higher discriminatory ability for Ps than our GRS-N did (AUC = 0.789 vs. 0.776, respectively) [25]. However, it should be emphasized that the association of *HLA-C* with Ps was significantly stronger in the study by Stawczyk-Macieja et al. than in our study (OR = 7.42 vs. OR = 3.98). Thus, this high predictive performance of GRS-Stawczyk-Macieja might be explained by the high discriminative power of *HLA-C*. We may speculate that larger effect size observed for *HLA-C* in the study by Stawczyk-Macieja as compared to our study may be due to a different age of onset. Two previous small-sample-size studies performed in a Polish population showed a strong association of *HLA-C* with juvenile Ps (OR = 18.73) and lack of association with late-onset Ps [51,52]. Unfortunately, the information on age of onset, as well as a proportion of patients with positive family history of Ps, is not provided in the study by Stawczyk-Macieja, making a reliable comparison with the present study impossible.

Chen et al. described a GRS (further named GRS-Chen) combining 10 SNPs in a population of European ancestry [23]. That study shared some similarities with the present study, i.e. ethnicity (European) and similar age of onset (24.6 years vs. 26.27 years). However, the proportion of patients with positive family history of Ps was markedly lower in the present study as compared to that by Chen et al. (43.3% vs. 76.7%). Importantly, 8 out of 10 SNPs forming the GRS-Chen are included in our GRS (rs3212227 and rs2235617 are perfect proxies for rs3213094 and rs6125829, respectively). Chen et al. found that the AUC for *HLA-C* was slightly higher than AUC for the GRS without *HLA-C* (0.662 vs. 0.638) and significantly lower than AUC for the GRS with *HLA-C* (0.720). The OR for top vs. bottom GRS-Chen quartiles was 10.55 (*P*_*trend*_ = 2,15 x 10^−11^). The SNPs forming GRS-Chen were found to cover 11.6% of phenotypic variation. The predictive accuracy of GRS-Chen and the OR for top vs. bottom quartiles are markedly lower than these of GRS-N (0.720 vs. 0.776 and 10.55 vs. 12.29, respectively). It may be speculated that worse performance of GRS-Chen is due to lower discriminative power of *HLA-C* (0.662 vs. 0.694 in our study). This might be partly explained by the fact that Chen et al. used a different tag SNP for *HLA-C* (i.e. rs10484554, OR = 3.07). We used rs4406273 as a *HLA-C* tag SNP (OR = 3.98). To assess this possibility, we rebuilt GRS-N with the substitution of rs4406273 by rs10484554 − GRS-N(subst.). The AUC for GRS-N(subst.) was lower as compared to GRS-N (0.741 vs. 0.776) but still slightly higher than AUC for GRS-Chen (0.741 vs. 0.720). On the other hand, we should take into account the fact that the age of onset in the present study was higher and the proportion of patients with positive family history of Ps lower as compared to those presented by Chen et al. Adjustment for these parameters would certainly increase the difference in the discriminatory ability in favor of our GRS.

It should be emphasized that in all previous studies mentioned above the discriminatory ability of the GRS was determined mainly by *HLA-C* (AUC for *HLA-C* was significantly higher than AUC for the non-*HLA-C* SNPs). In contrast, in our study the discriminative power of the GRS-N(+)HLA(-) and GRS-HLA were similar, which suggests that the non-*HLA-C* SNPs from our study had larger influence on the discriminatory ability of the GRS than non-*HLA-C* SNP from previous studies.

The observation of markedly better discriminatory ability of the GRS based on the polymorphisms previously described in Ps in T1Ps patients than in T2Ps patients seems to be important for future analyses. This may be simply explained by a stronger genetic basis in T1Ps than in T2Ps. However, it may be also due to the fact that T1Ps is much more common than T2Ps. Thus, we can speculate that T2Ps patients were under-represented in Ps cohorts used in GWAS studies. If that is the case, a GWAS focusing on T2Ps patients might reveal the associations with novel loci.

Several limitations to this study need to be acknowledged. First, we did not include all known psoriasis loci in the analysis. Second, our study had a limited power to detect the associations with several SNPs, especially with low MAF (S1 Table). Thus, we used the combined weighted GRS as the major genetic variable examined to overcome the problem of low power resulting from the inclusion of rare variants in the analysis. Third, our study was prone to overfitting (similarly to studies by Chen et al. and by Stawczyk-Macieja et al. [23,25]). However, it should be emphasized that all SNPs included in our top GRS (GRS-N) had a well-documented (meta-analysis or large-cohort studies) association with Ps/PsA in Caucasian populations, which reduces the risk of overfitting of the cumulative model due to potential false-positive findings in our single dataset. Additionally, we performed an internal validation, which showed a similar discriminatory ability of the model in Training and Test Sets. However, further validation on independent cohorts is needed to confirm our findings.

In summary, we confirmed an association of Ps with several previously identified genetic risk factors in a Polish population. We also found that a GRS combining 16 SNPs at least nominally associated with Ps in our population (GRS-N) had a significantly better discriminatory ability than *HLA-*C or GRS combining SNPs associated with Ps after the Bonferroni correction (GRS-B). In contrast, adding additional SNPs to the GRS did not increase the discriminative power significantly. The total risk conferred by the GRS-N seems to be higher than the risk conferred by another GRS described in a Caucasian population [23] and the 16 SNPs forming GRS-N covered almost 20% of phenotypic variation. We demonstrated that GRS-N was associated with age of onset and family history of Ps.

## Supporting information

S1 Table SNPs selected for analysis. **When OR for combined analysis was not provided in the reference, an OR for discovery sample is given. In case of SNPs not fulfilling selection criteria the reason for inclusion in the study is given in “Remarks”.** OR^ref^- odds ratio in previous studies; OR^pres^- odds ratio in the present study; *P*^ref^- *P* value in previous studies; RAF^ref^- risk allele frequency in the controls in previous studies; RAF^pres^- risk allele frequency in the controls in the present study; ^§^Statistical power of our study to detect the association with an alpha of 0.05 (based on risk allele frequency in our control group and OR from the reference study); OR^D^- odds ratio for discovery sample; ^#^OR for psoriatic arthritis; *based on OR in German population.
(DOCX)

S2 Table SNPs forming particular genetic risk scores. (DOCX)

S3 Table SNPs associations with psoriasis, type I psoriasis and type II psoriasis. CI- confidence interval; OR- odds ratio; RAF- risk allele frequency.
(DOCX)

S4 Table SNPs associations with PsC and PsA. CI- confidence interval; OR- odds ratio; PsC- purely cutaneous psoriasis; PsA- psoriatic arthritis; RAF- risk allele frequency.
(DOCX)

S5 Table Predictive performance of GRS-N. PV: predictive value.
(DOCX)

S6 Table Comparison of previous studies reporting GRSs in Ps with the present study. ^*^rs1265181 is in complete LD (r^2^ = 1.0) with rs4406273 in Han Chinese population. ^#^rs4406273 can be used as a substitute for genotyping of HLA-C*06:*02* in people of European, Pakistani, Thai, Chinese, or Japanese ancestry; LD was very strong between rs4406273 and HLA-C*06:*02* in four populations of European descent from the United States, Finland, Great Britain, and Italy (r^2^ = 0.984), and in three Asian populations from Japan and China (r^2^ = 1.000) [43].
(DOCX)
