# Supplementary material for: The association between 38 previously reported polymorphisms and psoriasis in a Polish population: High predicative accuracy of a genetic risk score combining 16 loci
Source: PLoS One. 2017 Jun 15;12(6):e0179348. doi: 10.1371/journal.pone.0179348 (PMC5472287; doi:10.1371/journal.pone.0179348)
Supplement: S1 Table — When OR for combined analysis was not provided in the reference, an OR for discovery sample is given. In case of SNPs not fulfilling selection criteria the reason for inclusion in the study is given in “Remarks”. ORref- odds ratio in previous studies; ORpres- odds ratio in the present study; Pref- P value in previous studies; RAFref- risk allele frequency in the controls in previous studies; RAFpres- risk allele frequency in the controls in the present study; §Statistical power of our study to detect the association with an alpha of 0.05 (based on risk allele frequency in our control group and OR from the reference study); ORD- odds ratio for discovery sample; #OR for psoriatic arthritis; *based on OR in German population. (DOCX) [file pone.0179348.s001.docx]

**S1 Table. SNPs selected for analysis. When OR for combined analysis was not provided in the reference, an OR for discovery sample is given. In case of SNPs not fulfilling selection criteria the reason for inclusion in the study is given in “Remarks”.**

| SNP | Gene | Risk allele | RAF^ref^ | RAF^pres^ | OR^ref^ | *P*^ref^ | OR^pres^ | Power^§^ | References | Remarks |
| --- | --- | --- | --- | --- | --- | --- | --- | --- | --- | --- |
| rs7552167 | *IL28RA* | G | 0.858 | 0.833 | 1.21 | 8.5 x 10^-12^ | 1.36 | 0.45 | 28 |  |
| rs7530511 | *IL23R* | C | 0.870 | 0.851 | 1.29 | 3.9 x 10^-3^ | 1.13 | 0.62 | 29 |  |
| rs11209026 | *IL23R* | G | 0.932 | 0.960 | 1.61 | 3.8 x 10^-4^ | 1.98 | 0.53 | 29 | rs11209026 is independent of rs7530511 (r^2^=0.007) and forms with rs7530511 a common risk haplotype [29] |
| rs2476601 | *PTPN22* | G | 0.881 | 0.859 | 1.40^#^ | 1.3 x 10^-2^ | 1.10 | 0.78 | 30 | rs2476601 is associated with several autoinflammatory diseases [Zheng J Petersen F, Yu X. The role of PTPN22 in autoimmunity: learning from mice. Autoimmun Rev. 2014; 13: 266-271. doi: 10.1016/j.autrev.2013.10.011] |
| rs4112788 | *LCE3C-LCE3B* | C | 0.642 | 0.627 | 1.21 | 1.4 x 10^-12^ | 1.19 | 0.65 | 31 |  |
| rs6701216 | *LCE1C* | T | 0.127 | 0.147 | 1.45 | 5 x 10^-5^ | 1.18 | 0.93 | 32 |  |
| rs702873 | *REL* | G | 0.560 | 0.557 | 1.23^D^ | 1.32 x 10^-7^ | 1.42 | 0.74 | 33 |  |
| rs10865331 | *B3GNT2* | A | 0.374 | 0.400 | 1.12 | 4.7 x 10^-10^ | 1.32 | 0.35 | 28 | Associated with ankylosing spondylitis [44] |
| rs17716942 | *IFIH1* | T | 0.860 | 0.893 | 1.38^D^ | 4.05 x 10^-8^ | 1.07 | 0.50 | 33 |  |
| rs30187 | *ERAP1* | T | Unk | 0.313 | 1.27 | 1.5 x 10^-9^ | 1.15 | 0.82 | 33 |  |
| rs20541 | *IL13* | C | 0.790 | 0.739 | 1.27 | 5 x 10^-15^ | 1.27 | 0.72 | 34 |  |
| rs1024995 | *TNIP1* | C | Unk | 0.137 | 1.27 | 3.92 x 10^-5^ | 1.26 | 0.62 | 33 |  |
| rs3212227 | *IL12B* | A | 0.785 | 0.774 | 1.56 | 1.7 x 10^-15^ | 1.69 | 0.99 | 29 |  |
| rs6887695 | *IL12B* | G | 0.679 | 0.697 | 1.42 | 2.7 x 10^-15^ | 1.38 | 0.96 | 29 | rs6887695 is in weak LD with rs3212227; both SNPs form a common risk haplotype [29] |
| rs2431697 | *PTTG1* | C | 0.177 | 0.396 | 1.20 | 1.11 x 10^-8^ | 1.15 | 0.64 | 35 |  |
| rs6908425 | *CDKAL1* | C | 0.767 | 0.775 | 1.26 | 4 x 10^-6^ | 1.24 | 0.66 | 36 |  |
| rs1150735 | *RNF39* | T | 0.325 | 0.344 | 1.59^#^ | 2.6 x 10^-6^ | 1.10 | 1 | 37 |  |
| rs1264569 | *TRIM39/RPP21* | A | 0.766 | 0.794 | 1.53^#^ | 2.7 x 10^-4^ | 1.75 | 0.97 | 37 |  |
| rs879882 | *POU5F1* | C | 0.627 | 0.615 | 1.63^#^ | 1.12 x 10^-6^ | 1.63 | 0.99 | 37 |  |
| rs4406273 | *HLA-C* | A | 0.092 | 0.112 | 4.32 | 4.5 x 10^-723^ | 3.98 | 1 | 28 |  |
| rs10484554 | *HLA-C* | T | 0.150 | 0.222 | 2.35 | 6.3 x 10^-45^ | 2.80 | 1 | 38 |  |
| rs13437088 | *MICA* | T | 0.260 | 0.303 | 1.32 | 9 x 10^-6^ | 1.57 | 0.86 | 39 |  |
| rs240993 | *TRAF3IP2* | T | 0.250 | 0.296 | 1.36^D^ | 8.7 x 10^-13^ | 1.11 | 0.94 | 33 |  |
| rs610604 | *TNFAIP3* | C | 0.320 | 0.305 | 1.19 | 9 x 10^-12^ | 1.09 | 0.57 | 34 |  |
| rs7007032 | *CSMD1* | T | 0.820 | 0.690 | 1.16 | 3.78 x 10^-8^ | 1.00 | 0.44 | 35 |  |
| rs1250546 | *ZMIZ1* | A | 0.579 | 0.595 | 1.10 | 6.8 x 10^-7^ | 1.12 | 0.27 | 28 | SNPs in strong LD are associated with Crohn disease; rs1250536 cause differential expression of *ZMIZ1* [40] |
| rs12580100 | *RPS26* | A | 0.868 | 0.896 | 1.17 | 1 x 10^-6^ | 1.14 | 0.27 | 41 |  |
| rs3751385 | *GJB2* | C | 0.521 | 0.833 | 1.14 | 8.57 x 10^-8^ | 1.07 | 0.40* | 35 | The association was replicated in German population (OR=1.19, *P*=7.93 x 10^-3^) [35] |
| rs7993214 | *COG6* | C | 0.649 | 0.622 | 1.40 | 2 x 10^-6^ | 1.03 | 0.97 | 32 |  |
| rs8016947 | *NFKBIA* | G | 0.570 | 0.531 | 1.19 | 4.3 x 10^-6^ | 1.27 | 0.88 | 28 |  |
| rs4780355 | *SOCS1* | T | Unk | 0.668 | 1.15 | 5.3 x 10^-7^ | 1.06 | 0.43 | 40 |  |
| rs12445568 | *FBXL19* | C | 0.368 | 0.406 | 1.16 | 1.2 x 10^-16^ | 1.09 | 0.50 | 28 |  |
| rs4795067 | *NOS2* | G | 0.351 | 0.347 | 1.20 | 4 x 10^-11^ | 1.21 | 0.63 | 41 |  |
| rs744166 | *STAT3* | C | 0.399 | 0.370 | 1.35^#^ | 1.35 x 10^-3^ | 1.00 | 0.95 | 42 |  |
| rs12720356 | *TYK2* | T | 0.90 | 0.929 | 1.34^D^ | 9.25 x 10^-6^ | 1.49 | 0.46 | 33 |  |
| rs892085 | *IL3/CARM1* | T | 0.558 | 0.602 | 1.17 | 3.0 x 10^-17^ | 1.15 | 0.53 | 28 |  |
| rs9304742 | *ZNF816* | C | 0.361 | 0.320 | 1.14 | 7.6 x 10^-11^ | 1.09 | 0.80* | 35 | The association was replicated in German population (OR=1.26, *P*=3.55 x 10^-3^) [35] |
| rs1008953 | *SDC4* | G | 0.750 | 0.745 | 1.16 | 4.9 x 10^-3^ | 1.35 | 0.42 | 38 |  |
| rs2235617 | *RNF114* | G | unk | 0.522 | 1.20 | 1.65 x 10^-6^ | 1.20 | 0.67 | 38 |  |

OR^ref^- odds ratio in previous studies; OR^pres^- odds ratio in the present study; *P*^ref^- *P* value in previous studies; RAF^ref^- risk allele frequency in the controls in previous studies; RAF^pres^- risk allele frequency in the controls in the present study; ^§^Statistical power of our study to detect the association with an alpha of 0.05 (based on risk allele frequency in our control group and OR from the reference study); OR^D^- odds ratio for discovery sample; ^#^OR for psoriatic arthritis; *based on OR in German population.
