# Supplementary material for: The association between 38 previously reported polymorphisms and psoriasis in a Polish population: High predicative accuracy of a genetic risk score combining 16 loci
Source: PLoS One. 2017 Jun 15;12(6):e0179348. doi: 10.1371/journal.pone.0179348 (PMC5472287; doi:10.1371/journal.pone.0179348)
Supplement: S2 Table — (DOCX) [file pone.0179348.s002.docx]

**S2 Table. SNPs forming particular genetic risk scores.**

| SNP | GRS-ALL | GRS-0.1 | GRS-N | GRS-N(+)HLA(-) | GRS-B | GRS-HLA | GRS-N(subst.) |
| --- | --- | --- | --- | --- | --- | --- | --- |
| rs7552167 | + | + | + | + |  |  | **+** |
| rs7530511 | + |  |  |  |  |  |  |
| rs11209026 | + | + | + | + |  |  | **+** |
| rs2476601 | + |  |  |  |  |  |  |
| rs4112788 | + | + |  |  |  |  |  |
| rs6701216 | + |  |  |  |  |  |  |
| rs702873 | + | + | + | + | + |  | **+** |
| rs10865331 | + | + | + | + |  |  | **+** |
| rs17716942 | + |  |  |  |  |  |  |
| rs30187 | + |  |  |  |  |  |  |
| rs20541 | + | + | + | + |  |  | **+** |
| rs1024995 | + | + |  |  |  |  |  |
| rs3212227 | + | + | + | + | + |  | **+** |
| rs6887695 | + | + | + | + |  |  | **+** |
| rs2431697 | + |  |  |  |  |  |  |
| rs6908425 | + | + |  |  |  |  |  |
| rs1150735 | + |  |  |  |  |  |  |
| rs1264569 | + | + | + | + | + |  | **+** |
| rs879882 | + | + | + | + | + |  | **+** |
| rs4406273 | + | + | + |  | + | **+** |  |
| rs10484554 | + |  |  |  |  |  | **+** |
| rs13437088 | + | + | + | + | + |  | **+** |
| rs240993 | + |  |  |  |  |  |  |
| rs610604 | + |  |  |  |  |  |  |
| rs7007032 | + |  |  |  |  |  |  |
| rs12580100 | + |  |  |  |  |  |  |
| rs3751385 | + |  |  |  |  |  |  |
| rs7993214 | + |  |  |  |  |  |  |
| rs8016947 | + | + | + | + |  |  | **+** |
| rs4780355 | + |  |  |  |  |  |  |
| rs12445568 | + |  |  |  |  |  |  |
| rs4795067 | + | + | + | + |  |  | **+** |
| rs744166 | + |  |  |  |  |  |  |
| rs12720356 | + | + | + | + |  |  | **+** |
| rs892085 | + |  |  |  |  |  |  |
| rs9304742 | + |  |  |  |  |  |  |
| rs1008953 | + | + | + | + |  |  | **+** |
| rs2235617 | + | + | + | + |  |  | **+** |
