# Supplementary material for: The association between 38 previously reported polymorphisms and psoriasis in a Polish population: High predicative accuracy of a genetic risk score combining 16 loci
Source: PLoS One. 2017 Jun 15;12(6):e0179348. doi: 10.1371/journal.pone.0179348 (PMC5472287; doi:10.1371/journal.pone.0179348)
Supplement: S3 Table — CI- confidence interval; OR- odds ratio; RAF- risk allele frequency. (DOCX) [file pone.0179348.s003.docx]

**S3 Table. SNPs associations with psoriasis, type I psoriasis and type II psoriasis.**

|  | | | | | Psoriasis | | | Type I psoriasis | | | Type II psoriasis | | |
| --- | --- | --- | --- | --- | --- | --- | --- | --- | --- | --- | --- | --- | --- |
| SNP | Chromosomal localization | Gene | Risk allele | RAF_ctrl_ | RAF_cases_ | OR (95% CI) | *P* | RAF_cases_ | OR (95% CI) | *P* | RAF_cases_ | OR (95% CI) | *P* |
| rs7552167 | 1p36.11 | *IL28RA* | G | 0.833 | 0.871 | 1.36 (1.05-1.75) | **0.017** | 0.869 | 1.33 (1.01-1.75) | **0.035** | 0.879 | 1.46 (0.92-2.30) | 0.10 |
| rs7530511 | 1p31.3 | *IL23R* | C | 0.851 | 0.866 | 1.13 (0.87-1-46) | 0.37 | 0.857 | 1.04 (0.79-1.37) | 0.77 | 0.899 | 1.55 (0.95-2.55) | 0.075 |
| rs11209026 | 1p31.3 | *IL23R* | G | 0.960 | 0.979 | 1.98 (1.14-3.44) | **0.013** | 0.985 | 2.69 (1.37-5.28) | **2.4x10^-3^** | 0.960 | 0.99 (0.46-2.16) | 0.99 |
| rs2476601 | 1p13.2 | *PTPN22* | G | 0.859 | 0.869 | 1.10 (0.85-1.44) | 0.46 | 0.865 | 1.04 (0.79-1.38) | 0.76 | 0.894 | 1.38 (0.85-2.24) | 0.19 |
| rs4112788 | 1q21.3 | *LCE3C-LCE3B* | C | 0.627 | 0.667 | 1.19 (0.99-1.44) | 0.07 | 0.682 | 1.28 (1.04-1.56) | **0.018** | 0.611 | 0.93 (0.68-1.28) | 0.67 |
| rs6701216 | 1q21.3 | *LCE1C* | T | 0.147 | 0.168 | 1.18 (0.92-1.51) | 0.20 | 0.171 | 1.20 (0.93-1.56) | 0.17 | 0.157 | 1.08 (0.71-1.65) | 0.72 |
| rs702873 | 2p13-p12 | *REL* | G | 0.557 | 0.642 | 1.42 (1.18-1.71) | **2.5x10^-4^** | 0.631 | 1.36 (1.12-1.65) | **3x10^-3^** | 0.697 | 1.70 (1.23-2.35) | **1.3x10^-3^** |
| rs10865331 | 2p15 | *B3GNT2* | A | 0.400 | 0.467 | 1.32 (1.10-1.60) | **2.5x10^-3^** | 0.458 | 1.27 (1.05-1.54) | **0.014** | 0.500 | 1.50 (1.10-2.04) | **7.9x10^-3^** |
| rs17716942 | 2q24 | *IFIH1* | T | 0.893 | 0.898 | 1.07 (0.79-1.43) | 0.68 | 0.897 | 1.05 (0.77-1.43) | 0.78 | 0.904 | 1.13 (0.68-1.90) | 0.64 |
| rs30187 | 5q15 | *ERAP1* | T | 0.313 | 0.344 | 1.15 (0.95-1.40) | 0.14 | 0.355 | 1.21 (0.99-1.49) | 0.06 | 0.303 | 0.96 (0.69-1.33) | 0.79 |
| rs20541 | 5q31.1 | *IL13* | C | 0.739 | 0.782 | 1.27 (1.02-1.56) | **0.026** | 0.776 | 1.23 (0.98-1.55) | 0.06 | 0.798 | 1.39 (0.96-2.02) | 0.08 |
| rs1024995 | 5q33.1 | *TNIP1* | C | 0.137 | 0.166 | 1.26 (0.98-1.62) | 0.07 | 0.163 | 1.23 (0.94-1.61) | 0.13 | 0.177 | 1.36 (0.90-2.04) | 0.14 |
| rs3212227 | 5q33.3 | *IL12B* | A | 0.774 | 0.853 | 1.69 (1.34-2.14) | **1.2x10^-5^** | 0.848 | 1.63 (1.27-2.10) | **1.3x10^-4^** | 0.869 | 1.94 (1.25-3.00) | **2.7x10^-3^** |
| rs6887695 | 5q33.3 | *IL12B* | G | 0.697 | 0.760 | 1.38 (1.12-1.69) | **2.1x10^-3^** | 0.752 | 1.32 (1.06-1.64) | **0.013** | 0.791 | 1.64 (1.13-2.38) | **9.7x10^-3^** |
| rs2431697 | 5q33.3 | *PTTG1* | C | 0.396 | 0.430 | 1.15 (0.96-1.38) | 0.13 | 0.422 | 1.11 (0.92-1.35) | 0.28 | 0.459 | 1.26 (0.93-1.71) | 0.09 |
| rs6908425 | 6p22.3 | *CDKAL1* | C | 0.775 | 0.811 | 1.24 (0.99-1.55) | 0.051 | 0.811 | 1.25 (0.98-1.59) | 0.07 | 0.808 | 1.22 (0.83-1.79) | 0.29 |
| rs1150735 | 6p21.3 | *RNF39* | T | 0.344 | 0.367 | 1.10 (0.91-1.33) | 0.31 | 0.364 | 1.09 (0.89-1.33) | 0.41 | 0.379 | 1.16 (0.85-1.59) | 0.36 |
| rs1264569 | 6p21.3 | *TRIM39/RPP21* | A | 0.794 | 0.871 | 1.75 (1.37-2.24) | **1.1x10^-5^** | 0.865 | 1.66 (1.28-2.16) | **1.8x10^-4^** | 0.894 | 2.18 (1.35-3.52) | **1.3x10^-3^** |
| rs879882 | 6p21.31 | *POU5F1* | C | 0.615 | 0.723 | 1.63 (1.35-1.98) | **3.4x10^-7^** | 0.730 | 1.69 (1.37-20.1) | **4.3x10^-7^** | 0.697 | 1.44 (1.04-2.00) | **0.028** |
| rs4406273 | 6p21.33 | *HLA-B/HLA-C* | A | 0.112 | 0.335 | 3.98 (3.13-5.06) | **4.6x10^-33^** | 0.373 | 4.70 (3.67-6.02) | **3.3x10^-39^** | 0.197 | 1.94 (1.30-2.89) | **1.6x10^-3^** |
| rs10484554 | 6p21.33 | *HLA-C* | T | 0.222 | 0.444 | 2.80 (2.29-3.41) | **5x10^-26^** | 0.478 | 3.19 (2.60-3.94) | **2.1x10^-30^** | 0.323 | 1.67 (1.20-2.33) | **2.3x10^-3^** |
| rs13437088 | 6p21.33 | *MICA* | T | 0.303 | 0.404 | 1.57 (1.30-1.90) | **3.3x10^-6^** | 0.396 | 1.50 (1.23-1.84) | **8.6x10^-5^** | 0.444 | 1.84 (1.35-2.51) | **9.8x10^-5^** |
| rs240993 | 6q21 | *TRAF3IP2* | T | 0.296 | 0.319 | 1.11 (0.92-1.35) | 0.28 | 0.322 | 1.13 (0.92-1.39) | 0.26 | 0.309 | 1.06 (0.76-1.48) | 0.72 |
| rs610604 | 6p23.3 | *TNFAIP3* | C | 0.305 | 0.325 | 1.09 (0.90-1.33) | 0.37 | 0.325 | 1.10 (0.89-1.35) | 0.39 | 0.321 | 1.08 (0.78-1.50) | 0.65 |
| rs7007032 | 8p23.2 | *CSMD1* | T | 0.690 | 0.691 | 1.00 (0.82-1.22) | 0.99 | 0.694 | 1.01 (0.82-1.25) | 0.90 | 0.682 | 0.96 (0.69-1.33) | 0.81 |
| rs12580100 | 12q13 | *RPS26* | A | 0.839 | 0.856 | 1.14 (0.89-1.46) | 0.31 | 0.852 | 1.11 (0.85-1.44) | 0.46 | 0.869 | 1.27 (0.81-1.98) | 0.29 |
| rs3751385 | 13q11-q12 | *GJB2* | C | 0.833 | 0.843 | 1.07 (0.84-1.37) | 0.57 | 0.844 | 1.08 (0.83-1.41) | 0.55 | 0.838 | 1.04 (0.69-1.57) | 0.86 |
| rs7993214 | 13q13.3 | *COG6* | C | 0.622 | 0.628 | 1.03 (0.85-1.24) | 0.79 | 0.638 | 1.07 (0.88-1.31) | 0.50 | 0.591 | 0.88 (0.64-1.20) | 0.41 |
| rs8016947 | 14q13 | *NFKBIA* | G | 0.531 | 0.591 | 1.27 (1.06-1.53) | **7.5x10^-3^** | 0.600 | 1.33 (1.09-1.61) | **3.6x10^-3^** | 0.556 | 1.10 (0.81-1.50) | 0.52 |
| rs4780355 | 16p13.13 | *SOCS1* | T | 0.668 | 0.680 | 1.06 (0.87-1.28) | 0.58 | 0.678 | 1.05 (0.85-1.28) | 0.66 | 0.687 | 1.09 (0.78-1.51) | 0.61 |
| rs12445568 | 16p11.2 | *FBXL19* | C | 0.406 | 0.427 | 1.09 (0.91-1.31) | 0.35 | 0.433 | 1.12 (0.91-1.36) | 0.26 | 0.404 | 0.99 (0.73-1.35) | 0.96 |
| rs4795067 | 17q11.2 | *NOS2* | G | 0.347 | 0.392 | 1.21 (1.01-1.46) | **0.049** | 0.397 | 1.24 (1.01-1.51) | **0.04** | 0.374 | 1.12 (0.82-1.54) | 0.49 |
| rs744166 | 17q21.31 | *STAT3* | C | 0.370 | 0.371 | 1.00 (0.83-1.21) | 0.97 | 0.386 | 1.07 (0.88-1.30) | 0.50 | 0.318 | 0.79 (0.57-1.10) | 0.15 |
| rs12720356 | 19p13.2 | *TYK2* | T | 0.929 | 0.951 | 1.49 (1.01-2.18) | **0.042** | 0.954 | 1.59 (1.04-2.44) | **0.03** | 0.939 | 1.19 (0.63-2.24) | 0.59 |
| rs892085 | 19p13.2 | *IL3/CARM1* | T | 0.602 | 0.634 | 1.15 (0.95-1.38) | 0.15 | 0.625 | 1.10 (0.90-1.34) | 0.35 | 0.668 | 1.33 (0.96-1.84) | 0.08 |
| rs9304742 | 19q13.41 | *ZNF816* | C | 0.320 | 0.339 | 1.09 (0.90-1.32) | 0.36 | 0.331 | 1.05 (0.86-1.29) | 0.62 | 0.369 | 1.07 (0.77-1.49) | 0.17 |
| rs1008953 | 20q12 | *SDC4* | G | 0.745 | 0.798 | 1.35 (1.09-1.68) | **5.9x10^-3^** | 0.803 | 1.39 (1.10-1.76) | **5.5x10^-3^** | 0.781 | 1.22 (0.84-1.75) | 0.29 |
| rs2235617 | 20q13.13 | *RNF114* | G | 0.522 | 0.568 | 1.20 (1.00-1.44) | **0.045** | 0.561 | 1.17 (0.97-1.42) | 0.11 | 0.591 | 1.32 (0.97-1.80) | 0.07 |

CI- confidence interval; OR- odds ratio; RAF- risk allele frequency
