# Supplementary material for: The association between 38 previously reported polymorphisms and psoriasis in a Polish population: High predicative accuracy of a genetic risk score combining 16 loci
Source: PLoS One. 2017 Jun 15;12(6):e0179348. doi: 10.1371/journal.pone.0179348 (PMC5472287; doi:10.1371/journal.pone.0179348)
Supplement: S4 Table — CI- confidence interval; OR- odds ratio; PsC- purely cutaneous psoriasis; PsA- psoriatic arthritis; RAF- risk allele frequency. (DOCX) [file pone.0179348.s004.docx]

**S4 Table. SNPs associations with PsC and PsA.**

|  | | | PsC | | | PsA | | |
| --- | --- | --- | --- | --- | --- | --- | --- | --- |
| SNP | Risk allele | RAF_ctrl_ | RAF | OR (95% CI) | *P* | RAF | OR (95% CI) | *P* |
| rs7552167 | G | 0.833 | 0.876 | 1.42 (1.06-1.90) | **0.018** | 0.860 | 1.24 (0.86-1.78) | 0.24 |
| rs7530511 | C | 0.851 | 0.861 | 1.08 (0.81-1.44) | 0.60 | 0.880 | 1.28 (0.87-1.88) | 0.20 |
| rs11209026 | G | 0.960 | 0.977 | 1.76 (0.95-3.27) | 0.063 | 0.984 | 2.53 (1.00-6.48) | **0.041** |
| rs2476601 | G | 0.859 | 0.874 | 1.13 (0.84-1.53) | 0.41 | 0.864 | 1.04 (0.71-1.50) | 0.85 |
| rs4112788 | C | 0.627 | 0.668 | 1.20 (0.97-1.49) | 0.09 | 0.668 | 1.20 (0.92-1.57) | 0.19 |
| rs6701216 | T | 0.147 | 0.175 | 1.24 (0.94-1.63) | 0.13 | 0.156 | 1.07 (0.75-1.53) | 0.69 |
| rs702873 | G | 0.557 | 0.649 | 1.47 (1.19-1.81) | **5x10^-4^** | 0.627 | 1.33 (1.03-1.73) | **0.032** |
| rs10865331 | A | 0.400 | 0.483 | 1.41 (1.15-1.72) | **7.8x10^-4^** | 0.435 | 1.16 (0.89-1.50) | 0.27 |
| rs17716942 | T | 0.893 | 0.894 | 1.02 (0.73-1.41) | 0.93 | 0.909 | 1.20 (0.78-1.86) | 0.41 |
| rs30187 | T | 0.313 | 0.361 | 1.24 (1.00-1.54) | **0.044** | 0.312 | 1.00 (0.76-1.31) | 0.98 |
| rs20541 | C | 0.739 | 0.773 | 1.20 (0.95-1.52) | 0.13 | 0.797 | 1.39 (1.01-1.90) | 0.036 |
| rs1024995 | C | 0.137 | 0.169 | 1.28 (0.97-1.70) | 0.08 | 0.162 | 1.23 (0.86-1.74) | 0.26 |
| rs3212227 | A | 0.774 | 0.859 | 1.79 (1.36-2.35) | **2.5x10^-5^** | 0.838 | 1.51 (1.08-2.12) | **0.017** |
| rs6887695 | G | 0.697 | 0.778 | 1.52 (1.20-1.93) | **5.1x10^-4^** | 0.729 | 1.17 (0.88-1.55) | 0.30 |
| rs2431697 | C | 0.396 | 0.455 | 1.28 (1.04-1.56) | **0.02** | 0.382 | 0.94 (0.73-1.73) | 0.67 |
| rs6908425 | C | 0.775 | 0.814 | 1.27 (0.99-1.64) | 0.058 | 0.808 | 1.22 (0.89-1.69) | 0.21 |
| rs1150735 | T | 0.344 | 0.354 | 1.04 (0.85-1.29) | 0.69 | 0.390 | 1.22 (0.93-1.58) | 0.14 |
| rs1264569 | A | 0.794 | 0.864 | 1.65 (1.25-2.18) | **5.3x10^-4^** | 0.886 | 2.02 (1.38-2.97) | **3x10^-4^** |
| rs879882 | C | 0.615 | 0.735 | 1.68 (1.35-2.09) | **6.1x10^-7^** | 0.705 | 1.49 (1.13-1.97) | **3.8x10^-3^** |
| rs4406273 | A | 0.112 | 0.341 | 4.09 (3.15-5.29) | **8.5x10^-29^** | 0.325 | 3.79 (2.79-5.17) | **2.4x10^-18^** |
| rs10484554 | T | 0.222 | 0.449 | 2.84 (2.28-3.54) | **8.5x10^-29^** | 0.435 | 2.69 (2.06-3.53) | **1.4x10^-13^** |
| rs13437088 | T | 0.303 | 0.407 | 1.58 (1.28-1.95) | **3x10^-5^** | 0.396 | 1.51 (1.16-1.97) | **2x10^-3^** |
| rs240993 | T | 0.296 | 0.322 | 1.13 (0.91-1.41) | 0.28 | 0.314 | 1.09 (0.82-1.43) | 0.56 |
| rs610604 | C | 0.305 | 0.324 | 1.09 (0.88-1.35) | 0.45 | 0.330 | 1.12 (0.85-1.47) | 0.41 |
| rs7007032 | T | 0.690 | 0.707 | 1.08 (0.87-1.35) | 0.50 | 0.662 | 0.88 (0.67-1.15) | 0.36 |
| rs12580100 | A | 0.839 | 0.853 | 1.11 (0.84-1.47) | 0.47 | 0.864 | 1.21 (0.84-1.75) | 0.30 |
| rs3751385 | C | 0.833 | 0.844 | 1.09 (0.82-1.43) | 0.56 | 0.838 | 1.03 (0.73-1.46) | 0.86 |
| rs7993214 | C | 0.622 | 0.609 | 0.95 (0.77-1.17) | 0.61 | 0.663 | 1.20 (0.91-1.57) | 0.19 |
| rs8016947 | G | 0.531 | 0.589 | 1.26 (1.03-1.55) | **0.019** | 0.594 | 1.29 (1.00-1.67) | **0.048** |
| rs4780355 | T | 0.668 | 0.677 | 1.04 (0.84-1.29) | 0.71 | 0.682 | 1.06 (0.81-1.40) | 0.66 |
| rs12445568 | C | 0.406 | 0.407 | 1.00 (0.82-1.23) | 0.97 | 0.468 | 1.28 (0.99-1.66) | 0.053 |
| rs4795067 | G | 0.347 | 0.401 | 1.26 (1.02-1.55) | **0.035** | 0.380 | 1.15 (0.88-1.50) | 0.31 |
| rs744166 | C | 0.370 | 0.371 | 1.00 (0.81-1.23) | 0.99 | 0.373 | 1.01 (0.78-1.32) | 0.92 |
| rs12720356 | T | 0.929 | 0.947 | 1.37 (0.89-2.11) | 0.15 | 0.961 | 1.89 (1.01-3.54) | **0.043** |
| rs892085 | T | 0.602 | 0.603 | 1.01 (0.82-1.24) | 0.96 | 0.693 | 1.49 (1.13-1.96) | **4.8x10^-3^** |
| rs9304742 | C | 0.320 | 0.344 | 1.12 (0.90-1.38) | 0.31 | 0.330 | 1.05 (0.80-1.38) | 0.73 |
| rs1008953 | G | 0.745 | 0.812 | 1.47 (1.15-1.89) | **2.1x10^-3^** | 0.769 | 1.14 (0.84-1.54) | 0.39 |
| rs2235617 | G | 0.522 | 0.599 | 1.37 (1.12-1.68) | **2.6x10^-3^** | 0.506 | 0.94 (0.73-1.21) | 0.63 |

CI- confidence interval; OR- odds ratio; PsC- purely cutaneous psoriasis; PsA- psoriatic arthritis; RAF- risk allele frequency
