# Supplementary material for: The association between 38 previously reported polymorphisms and psoriasis in a Polish population: High predicative accuracy of a genetic risk score combining 16 loci
Source: PLoS One. 2017 Jun 15;12(6):e0179348. doi: 10.1371/journal.pone.0179348 (PMC5472287; doi:10.1371/journal.pone.0179348)
Supplement: S5 Table — PV: predictive value. (DOCX) [file pone.0179348.s005.docx]

**S5 Table. Predictive performance of GRS-N.**

| Dataset | Accuracy | Sensitivity | Specificity | Positive PV | Negative PV |
| --- | --- | --- | --- | --- | --- |
| Training Set (75%) | 71.6% | 70.2% | 73.0% | 72.0% | 71.2% |
| Test Set (25%) | 73.3% | 69.0% | 77.2% | 72.9% | 73.7% |
| Overall | 72.0% | 69.9% | 74.1% | 72.2% | 71.8% |

PV: predictive value.
