# Supplementary material for: The association between 38 previously reported polymorphisms and psoriasis in a Polish population: High predicative accuracy of a genetic risk score combining 16 loci
Source: PLoS One. 2017 Jun 15;12(6):e0179348. doi: 10.1371/journal.pone.0179348 (PMC5472287; doi:10.1371/journal.pone.0179348)
Supplement: S6 Table — *rs1265181 is in complete LD (r2 = 1.0) with rs4406273 in Han Chinese population. #rs4406273 can be used as a substitute for genotyping of HLA-C*06:02 in people of European, Pakistani, Thai, Chinese, or Japanese ancestry; LD was very strong between rs4406273 and HLA-C*06:02 in four populations of European descent from the United States, Finland, Great Britain, and Italy (r2 = 0.984), and in three Asian populations from Japan and China (r2 = 1.000) [43]. (DOCX) [file pone.0179348.s006.docx]

**S6 Table. Comparison of previous studies reporting GRSs in Ps with the present study.**

|  | Chen et al. [23] | Stawczyk-Macieja et al. [25] | Yin et al. [26] | Present study |
| --- | --- | --- | --- | --- |
| Population | European ancestry | Polish from Northern Poland | Chinese | Polish |
| Study group | Psoriasis | Plaque psoriasis diagnosed by dermatologist | Psoriasis diagnosed by at least 2 dermatologist | Psoriasis diagnosed by dermatologist |
| No of Cases | 731 | 148 | 4541 | 480 |
| Control group |  |  | Healthy individuals with no family history of autoimmune disorders | General population |
| No of Controls | 2084 | 146 | 4278 | 490 |
| Age of onset | 24.6 | Unavailable | 21.31 | 26.27 |
| Positive family history of Ps | 76.7% | Unavailable | 31.34% | 43.3% |
| Genotyping method for *HLA-Cw*0602* | rs10484554 | Direct genotyping | rs1265181**^*^** | rs4406273^#^  rs10484554 |
| *HLA-Cw*0602* frequency in controls | 0.158 | Unavailable | 0.284 | 0.112 (rs4406273)  0.222 (rs10484554) |
| *HLA-Cw*0602* frequency in cases | 0.351 | Unavailable | Unavailable | 0.335 (rs4406273)  0.444 (rs10484554) |
| OR for *HLA-Cw*0602* | 3.07 | 7.42 | 21.96 | 3.98 (rs4406273)  2.80 (rs10484554) |

^*^rs1265181 is in complete LD (r^2^=1.0) with rs4406273 in Han Chinese population. ^#^rs4406273 can be used as a substitute for genotyping of *HLA-C*06:02* in people of European, Pakistani, Thai, Chinese, or Japanese ancestry; LD was very strong between rs4406273 and *HLA-C*06:02* in four populations of European descent from the United States, Finland, Great Britain, and Italy (r^2^=0.984), and in three Asian populations from Japan and China (r^2^=1.000) [43]
